# Assessment of Vaccine-Induced Immunity Against Canine Visceral Leishmaniasis

**DOI:** 10.3389/fvets.2019.00168

**Published:** 2019-06-04

**Authors:** Javier Moreno

**Affiliations:** WHO Collaborating Centre for Leishmaniasis, Laboratory for Reference and Research in Parasitology, Centro Nacional de Microbiología, Instituto de Salud Carlos III, Majadahonda, Spain

**Keywords:** vaccine, canine visceral leishmaniais, immunity, efficacy, *Leishmania infantum*

## Abstract

Canine visceral leishmaniasis is an increasingly important public health problem. Dogs infected by *Leishmania infantum* are the main domestic reservoir of the parasite and play a key role in its transmission to humans. Recent findings have helped in the development of novel diagnostic methods, and of control measures such as vaccines, some of which are already commercially available. However, quantitative procedures should be followed to confirm whether these vaccines elicit a cell-mediated immune response. The present work describes the need for this evaluation, and the techniques available for confirming this type of immune response.

## Zoonotic Visceral Leishmaniasis: a Growing Public Health Concern

Zoonotic visceral leishmaniasis (ZVL) is a vector-borne disease caused by the protozoan parasite *Leishmania infantum* (syn. *Leishmania chagasi*). In the Old World the parasite is transmitted by the bite of sand flies belonging to the genus *Phlebotomus*; in the New World the members of the genus *Lutzomyia* takes on this role. ZVL occurs in Mediterranean Europe, in North Africa and the Near East, Central Asia, China and Latin America, appearing in foci that coincide precisely with the geographical distribution of the disease vectors. The incidence of human visceral leishmaniasis (HVL) is estimated at 4,500–6,800 cases in the Americas, 1,200–2,000 in Mediterranean countries, and 5,000–10,000 across the Middle East to Central Asia (1).

Dogs, the main reservoir of the parasite, are susceptible to canine visceral leishmaniasis (CVL) (Box 1). The seroprevalence of *Leishmania* for the canine population ranges from 3 to 30% depending on the area and ecological variables (2). It is higher in areas where transmission can occur throughout the year; for example, in the south of Bahia, Brazil, it may be as high as 50.3% (3). However, when PCR-based tools are used for screening, prevalence figure can be even 3 times higher than that detected by serology (4). Indeed, follow-up studies of dogs living in areas where active transmission occurs show virtually all of them to have been in contact with the parasite at some point in their lives (5).

Box 1 **Main clinical characteristics and symptoms of canine visceral leishmaniasis.**

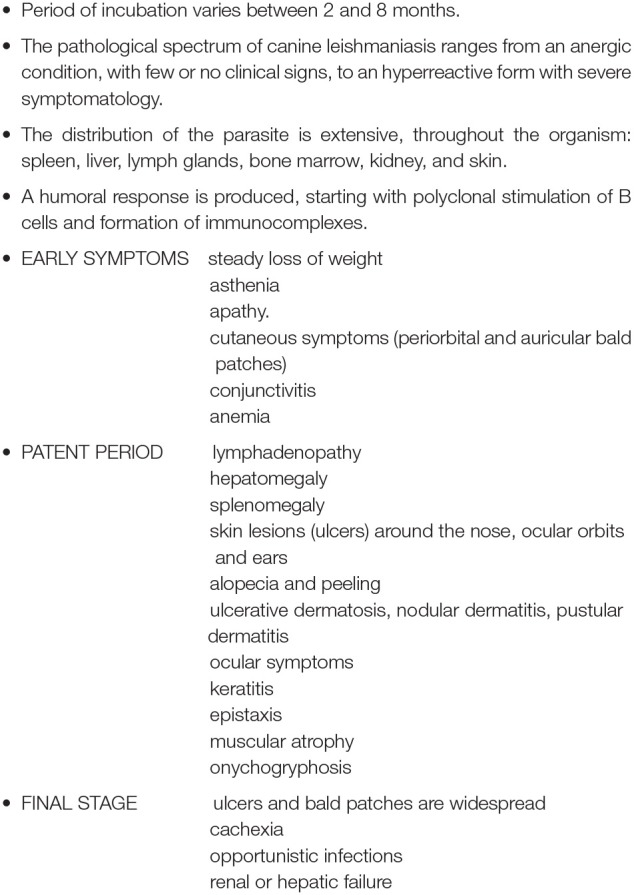

Wild animals such as wolves, jackals and foxes, hares and rabbits have also been described to act as reservoirs of the parasite. However, the proximity of dogs to humans, the high prevalence of infection among them, and the ease with which they transmit the parasite to sand flies, allow for the domestic transmission of *L. infantum* to humans. Actually, several studies have reported a correlation between the incidence of CVL and HVL (6).

CVL is not, therefore, only an important veterinary problem; it is also a major public health concern (7, 8). Infected dogs are directly involved in the spread of ZVL to disease-free areas. The appearance of cases of human leishmaniasis in previously non-endemic areas is usually preceded by the appearance of competent vectors and cases of CVL; such has been reported from both northern Italy (9, 10) and northern Argentina (11). The recent PAHO/WHO report on the human leishmaniasis situation in the Americas indicates that while the total number of cases of cutaneous leishmaniasis has remained stable over recent years, the number of cases of HVL has increased by 26.4% in the region, with increases in the fatality rate and number of deaths seen since 2014 (12). The transmission of HVL is increasing in Brazil, where the age-standardized disability-adjusted life years values associated with the disease increased by 83.6% between 1990 and 2016, and the age-standardized incidence rate and the years of life lost increased by 52.9 and 108% respectively over the same period (13).

Preventing the expansion of ZVL and disease transmission to humans requires surveillance of the vector, the implementation of measures to reduce the prevalence and incidence of CVL, and the development of procedures for assessing the impact of such control measures in affected populations.

## Prevention of *L. infantum* Infection in Dogs

The natural history of CVL is complex and depends on multiple factors like nutritional and immunological state of the animal, age, dog breed, or virulence of the parasite (14). The result is a dynamic spectrum of naturally infected dogs ranging from resistant, asymptomatic animals to those with severe disease (15). The number of *Leishmania-*infected dogs is much higher than the number that actually develops the disease; as a result, the overall burden of infection in the canine population in endemic areas is unknown (16). Some of these *Leishmania*-infected asymptomatic animals may act as “carriers,” especially if they harbor parasites in the skin; some 51% of the dogs from endemic areas are PCR skin-positive (17). The existence of such a significant source of parasites hinders the implementation of effective control measures. Certainly, strategies involving drug treatment for CVL have been shown unsuccessful; chemotherapy does not clear parasites from dogs, and although the majority improve clinically, relapses are frequent and the animals remain infectious to sand flies (5). Eliminating seropositive dogs does not help either; not only is it ethically unacceptable, it has been found to have no influence on disease prevalence (18, 19).

In this complex scenario, the best option for the efficient control of CVL is prevention, both of sand flies biting dogs (20), and of dogs developing the disease (21). Several insecticides and repellents are available in the form of collars, lotions and pipettes that impregnate the animal's skin and prevent sand fly bites. Controlled trials of these products have returned good results (22), but their effectiveness may decrease if dog owners fail to maintain their use (23).

Vaccines against CVL can provide dogs with specific, internal protection against developing clinical disease. Strong, specific and permanent immunity can be induced, preventing the multiplication and dispersion of the parasite, ruling out the development of CVL. The prophylactic vaccines currently available represent a clear advance in the control of this disease. Given their ease of use, and their cost/benefit ratio, prophylactic vaccines are usually the most effective prevention and control tools at our disposal (15).

### Development of Vaccines for CVL: A Challenging Task

Parasites are complex eukaryotic unicellular and multicellular pathogens. Most have very complicated life cycles that include the infection of intermediate invertebrate hosts; their morphological and genetic complexity makes them challenging targets for vaccines; and the parasites have evolved to resist the host immune response by evading effectors or preventing their production (24).

**Figure 1:**
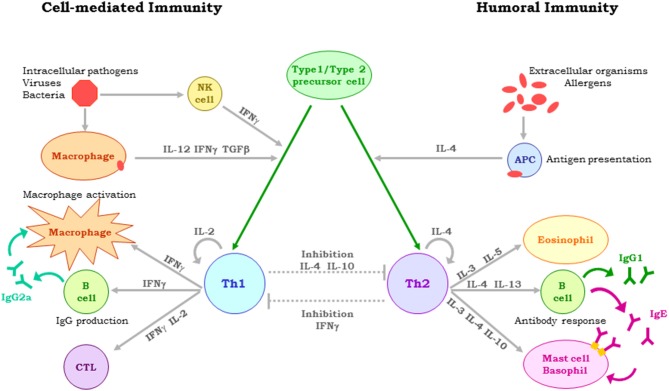
Dichotomy of the immune reaction to *Leishmania*, determined by the type of CD4 + T-cells and cytokines involved. In naturally infected dogs, resistance or susceptibility to CVL depends on the immune response elicited. Protection is associated with Th1 cell-mediated immunity, with IL-2, TNF-α, and IFN- γ stimulating the leishmanicidal activity of macrophages. Susceptibility is associated with a Th2 response and high antibody titres.

Early studies showed that parasites may trigger immediate-type hypersensitivity or delayed-type hypersensitivity, both mediated by CD4+ T helper (Th) cells (25). This reaction dichotomy was explained following the discovery that CD4+ T cells could be classified into multiple subsets depending on their cytokine expression profile, i.e., Th1 cells expressing interferon(IFN)-γ, interleukin(IL)-12 and/or tumor necrosis factor (TNF)-α (inducing delayed -type hypersensitivity), and Th2 cells expressing IL-4, IL-5, and IL-13 (promoting immediate-type hypersensitivity) (26). In the case of the *Leishmania* protozoan (an obligate intracellular parasite that infects mammalian host macrophages), this reaction dichotomy was demonstrated when BALB/c and C57BL6 mice experimentally infected with *Leishmania major* promastigotes developed either a Th2 or Th1 response associated respectively with either exacerbation or healing of the infection (27) (Figure 1). To prevent parasite multiplication and dissemination, an efficient cell-mediated immune response is required, involving dendritic cell-primed CD4+ (Th1 type) and CD8+ T lymphocytes that produce IFN-γ, and/or IL-12, and/or TNF-α. These cytokines activate infected macrophages to produce nitric oxide and reactive oxygen species, which lead to the physical elimination of the parasite (28). *Leishmania* has, however, evolved sophisticated mechanisms that help it prevent these responses (29). It also induces the expression of immunosuppressive IL-10, interferes with the production of *Leishmania*-specific antibodies, and stops the expansion of parasite-specific CD8-T cell clones, as well as disables the antigen presenting capacity of macrophages (30). Different high throughput techniques have shown that *Leishmania* infection affects the expression of a multitude of host genes (31), with the progress of the infection dependent on the balance struck between the virulence of the parasite and the host's innate and adaptive immune responses (32). The dichotomy in the reaction to *Leishmania* is seen in naturally infected dogs from the same disease-endemic area developing either severe CVL or remaining asymptomatic. The complexity of host-parasite interactions hinders the development of effective vaccines against CVL, certainly making it very difficult to identify a single hallmark of protection against leishmaniasis. All the factors involved in the response to infection by *Leishmania* must therefore be studied in detail (15, 33).

For many years, dogs were thought the most susceptible host in the transmission cycle, and that they had no possibility of recovery (14). This idea developed because of the high percentage of seropositive dogs that developed the disease, the large number of cases of CVL in endemic areas compared to HVL, and the only partial effectiveness of chemotherapy in sick animals. In contrast, when active HVL is successfully treated, cell-mediated immunity toward *Leishmania* spp. is developed (34, 35).

The observation that dogs naturally infected by *L. infantum* could actually develop a protective response to the disease was the proof of concept needed to show that the induction of immune protection against canine leishmaniasis was feasible (36). Several experimental infection trials later demonstrated that it was possible to induce this protective response experimentally (5). Nevertheless, obtaining an effective vaccine against CVL, capable of inducing a safe, strong and prolonged *Leishmania*-specific protective response in the dog, is a challenging task. Eliciting this type of cell-mediated response by vaccination is more difficult than obtaining a humoral response, especially given the antigenic complexity of the pathogen and its possession of evasion mechanisms (37).

Many strategies for inducing immunity against *Leishmania* infection have been tested in murine models (with greater or lesser success). These have been based on the use of killed *Leishmania* parasites, attenuated parasites, different antigen fractions, purified proteins, recombinant proteins, synthetic peptides, non-protein antigens, bacterial and virus-expressed parasite immunogens, and even “bare” parasite DNA (including the DNA of plasmids or linear vectors) (38–40). Only a few have been tried in dogs, however, because of the technical difficulties involved in handling the experimental animals and the high costs involved. Certainly, very few vaccine candidates for CVL have been tested in double-blind randomized field trials.

Partial protection against CVL has been reported after immunization with sonicated or autoclaved promastigotes (41, 42), with purified and recombinant *Leishmania* proteins (43–46), with parasite DNA (47, 48), and with attenuated *Leishmania* strains (49). A review summarizing the few efficacy studies performed in dogs, involving different types of *Leishmania* antigen (purified proteins, recombinant proteins or DNA), adjuvants and post-vaccination *Leishmania infantum* challenge, indicated different levels of protection to be obtained by the different vaccine candidates (50). Later, the attenuated *L. donovani* centrin-deleted strain [LdCen(-/-)] (when used as a vaccine) was found to reduce the parasite burden of subsequently infected dogs by up to 87.3% at 18 months post-challenge (51). The immunogenicity of, and partial protection afforded by, recombinant non-pathogenic *Leishmania tarentolae* expressing the A2 and cystein proteinases A and B proteins has also been reported (52). Alum-precipitated, autoclaved *Leishmania major* mixed with Bacillus Calmette-Guérin (BCG) and imiquimod was shown in a field trial to be of low efficacy in dogs (53) (Table 1).

**Table 1:** Vaccine trials for canine visceral leishmaniasis.

**Vaccine formulation**	**Type of trial**	**Cell immunity test**	**Efficacy**	**References**
*L. braziliensis* sonicated promastigotes + BCG	Experimental infection 2.3 × 10^6^ promastigotes IV	CPA	Partial -	(41)
Purified fucose mannose lingand + QuilA saponin	Phase III—Natural infection	LST	80.0%	(43)
Recombinant fusión protein Q	Experimental infection 500,000 promastigotes IV	LST	90%	(44)
Alum precipitated *L. major* autoclaved promastigotes + BCG	Phase III—Natural infection	LST	69.3%	(42)
Recombinant proteins H1, HASPB1 + Montanide. Polyprotein MML + MPL-SE	Experimental infection 1 × 10^8^ promastigotes IV	CPA	Partial	(45)
Plasmid with CPA and CPB —recombinant protein CPa, CPB + CpG ODN + Montanide. Prime—boost vaccination	Experimental infection 5 × 10^6^ promastigotes IV	CPA, LST Cytokine analysis	Not determined	(47)
DNA-LACK plasmid followed by rVaccinia virus containing the same gene (rVV-LACK). Prime-boost vaccination	Experimental infection 10^8^ promastigotes IV	CPA Cytokine analysis	Partial	(48)
Excreted/Secreted proteins + QA-21 saponin	Experimental infection 10^8.5^ promastigotes IV	CPA, ELISpot IFN- γ CMLA	Partial	(46)
Attenuated line L. infantum H-line), established by culturing promastigotes *in vitro* under gentamic in pressure	Phase III—Natural infection	None	93% (estimated)	(49)
Live attenuated *L. donovani* parasites LdCen(–/–)	Experimental infection 10^7^ promastigotes IV	CPA Cytokine analysis	Not determined	(51)
Alum precipitated autoclaved *L. major* mixed with BCG and imiquimod	Phase III—Natural infection	LST	40.4%	(53)
*L. tarentolae* expressing the A2 and cistein proteinases A and B proteins	Experimental infection 4 × 10^7^ *promastigotes IV*	LST Cytokine analysis	Partial	(52)

*CPA, Cell Prilferation Assay; LST, Leishmanin Skin Test; CMLA, Canine Macrophage Leishmanicidal Assay*.

To date, three vaccines against CVL have been approved, one in Brazil and two in Europe:

- Leish-Tec (Hertape Calier, Brazil), based on the recombinant protein A2, with saponin as an adjuvant (54, 55),
- Canileish (Virbac, France) made with *L. infantum* excreted/secreted antigens, with QA-21 as an adjuvant (56), and
- Letifend (Laboratorios Leti, Spain), based on the fusion protein Q, formulated without adjuvant (57).

The level of protection and efficacy in the prevention of disease reported for all three vaccines was similar (92–98 and 68–72%, respectively) (54, 58, 59) (Table 2). However, Leish-Tec was mainly assessed via the expression of induced antigen-specific IgG2 antibodies (60), while Letifend was assessed via the cellular immunity detected by the leishmanin skin test (57). In contrast, Canileish was found to induce a specific humoral response as well as specific cellular immunity, as confirmed by (i) the appearance of *Leishmania*-specific Th1 cell clones able to produce IFN-γ upon stimulation with leishmanial antigens, (ii) the induced leishmanicidal activity of macrophages, and (iii) the increased expression of iNOS and NO (which finally kills the parasite) (56). The specific cell-mediated immune response against the parasite was strong and remained effective against experimental challenge at 1 year (46).

**Table 2:** Comparison of CVL vaccines currently marketed.

**VACCINE (References)**	**LEISH-TEC (54)**	**CANILEISH (58)**	**LETIFEND (59)**
Vaccine formulation	A2 + saponin	*L. infantun* excreted/secreted protein + QA21	Q chimeric protein
Efficacy in the prevention of clinical signs	71.0%	68.4%	72%
Level of protection	96.4%	92.7%	98%
Reduction of symptoms	YES	YES	YES
Reduction of parasite burden after experimental infection (PCR)	YES	YES	YES
IgG2 expression	YES	YES	YES
Th1 cells activation	Not determined	YES	Not determined
IFN- γ expression after stimulation	YES	YES	Not determined
Leishmanicidal activity	Not determined	YES	Not determined
LST / DTH after infection	Not determined	YES	YES

### Assessing Immunity Against CVL

Usually, the assessment of the immunogenicity and efficacy of CVL vaccines has consisted of the clinical, serological and parasitological follow-up of vaccinated animals. However, several procedures can be followed to quantify the level of cell-mediated immunity (T cell memory) induced by natural infection and experimental immunization (Table 3). Most are based on the specific recognition of parasite antigens by *Leishmania*-specific T cell clones. Tests should be periodically re-performed, since in dogs the immune response to *Leishmania* can change (15).

**Table 3:** Methods for measuring cell-mediated immunity to *leishmania* infection or vaccination in dogs.

**METHOD**	**Context**	**Tissue/cell**	**Stimulant**	**Type of response detected**	**Parameter measured**	**Remark**
Leishmanin Skin Test- LST	*in-vivo*	Skin	Leishmanin-phenolized promastigotes	Delayed type hypersensitivity (DTH) response	Diameter of the intradermal reaction	- Previous exposure to *Leishmania* (epidemiological studies) - Efficacy trials
Cell Proliferation Assay—CPA	*in-vitro*	PBMC	-SLA -Purified proteins	Activation of *Leishmania*-specific T cell memory clones	Index of stimulation (DNA synthesis)	-Antigenicity of *Leishmania* protein -Immunogenicity of vaccine candidates - Efficacy trials
ELISpot—IFN- γ	*in-vitro*	PBMC	- SLA -Purified proteins	Activation of *Leishmania*-specific T cell memory clones producing IFN- γ	Number of spots	- Immunogenicity of vaccine candidates -Efficacy trials
Canine Macrophage Leishmanicidal Assay—CMLA	*in-vitro*	Macrophages and PBMCs	*Leishmania*-infected macrophages	Leishmanicidal activity induced by *Leishmania*-specific T cell memory clones	- Index of reduction of intracellular amastigotes -NO levels -iNOS expression	-*Leishmania*-specific functional CMI -Immunogenicity of vaccine candidates
Whole blood Assay—WBA	*Ex-vivo*	Peripheral blood	- SLA - Purified proteins	Profile of cytokines produced	Production of Th1 cytokines	-Previous exposure to *Leishmania* (epidemiological studies) - Antigenicity studies -Efficacy trials

*SLA, Soluble leishmanial antigen*;

*PBMC, Peripheral blood mononuclear cells*;

*CMI, Cell-mediated immunity*;

*NO, Nitric oxide*;

*iNOS, Inducible nitric oxide synthase*.

### The Leishmanin Skin Test—LST

This involves the inoculation of phenolized promastigotes into the epidermis and the measurement of the corresponding intradermal reaction—a delayed-type hypersensitive (DTH) response that can be examined under field conditions. This test provides a physiological means of assessing the development of *Leishmania*-specific cell-mediated immunity associated with the state of “resistance” to the parasite. In humans, the LST is a good detector of acquired protective immunity to *Leishmania*, becoming positive after effective therapy for HVL (35, 61). Further, the LST+ rate is inversely associated with the incidence of HVL (e.g., in populations with an LST+ rate of >45%, few cases of VL are seen).

LST is also a good method to assess anti-*Leishmania* specific DTH cellular responses in dogs, particularly under field conditions (62, 63). Most dogs that develop cell-mediated immunity do so early in infection, although some dogs with a positive LST result do develop clinical leishmaniasis (64, 65) (note that asymptomatic dogs show stronger LST+ reactions than do symptomatic ones). The LST+ reaction reflects a lack of progression of the disease, making it one of the most useful test for evaluating *Leishmania*-specific cell-mediated immunity (66–69). An LST+ reaction may indicate that the immune system is controlling the infection, even in animals that have a positive spleen culture (70). The use of LST in vaccine trials is limited to phases IIb and III, when it becomes necessary to confirm a *Leishmania*-specific cell-mediated response.

### The *in vitro* Cell Proliferation Assay—CPA

This technique involves the *in vitro* stimulation of peripheral blood mononuclear cells (PBMCs) with leishmanial soluble antigen to confirm the presence of circulating *Leishmania*-specific memory T cell clones. The degree of cell proliferation (measured via the synthesis of DNA in the culture, or by cell division), indicates the degree of specific cell-mediated immunity against the parasite. Such testing has been used to assess the antigenicity of *Leishmania* proteins in humans (71).

In dogs, the intensity of the CPA and LST responses are correlated (52, 72), and a *Leishmania*-specific CPA+ response is associated with recovery after treatment for CVL. Relapse in treated animals is associated with the lack of, or the disappearance of, a positive CPA result (73).

Unlike the LST, *in vitro* cell stimulation is a very useful way of assaying the antigenicity of vaccine candidate proteins, and for detecting specific cell responses after immunization. In experimentally infected dogs, the CPA has successfully been used to examine the antigenicity of different *Leishmania* proteins, such as P-8 (74) HSP-70, KMP-11, PFR-2 (75) and PSA (76). Stimulation with the specific proteins included in the vaccine formulation also allows one to determine their capacity to induce T cell memory clones (45, 56, 77–79), and to assess the duration of the immunity produced (80).

CPA can be complemented with the analysis of the cytokines (i.e., those involved in the Th1 response, but mainly IFN-γ, the effector cytokine involved in the activation of macrophages to kill the parasites) secreted into the supernatant, allowing for a better characterization of the cellular responses activated. CPA thus becomes an IFN-γ release assay (IGRA)—the type of cell assay used to demonstrate immunity to intracellular pathogens (51, 81–84).

### The ELISpot—IFN-γ Test

This is an IGRA-type test that combines the *in vitro* cell stimulation of PBMCs with the *in situ* expression of IFN-γ. This allows the direct quantification of the frequency of T cell clones producing IFN-γ after challenge with the leishmanial antigen—or vaccine stimulation—and therefore determines the potency of the immunity induced. This assay has proven useful for determining the immunological condition of *Leishmania*-infected people who are LST- (85). IFN-γ expression by *Leishmania*-specific T-cells is key in disease resistance. Several studies report the predominant role of IFN-γ in the activation of macrophages and the stimulation of their leishmanicidal activity in mice [reviewed in (26, 86, 87)]. The same role has been confirmed in dogs; high IFN-γ expression levels in peripheral blood lymphocytes from asymptomatic animals following stimulation with leishmanial antigen indicate a response to vaccination and are associated with the absence of symptoms (74, 88–90). All the latter studies emphasize the importance of T-cell-derived IFN-γ as a hallmark of immunity, and highlight the suitability of this approach when evaluating the efficacy of CVL vaccines. It is important to note that while the IFN-γ levels related to immunity to *Leishmania* are derived from T cells, the provenance of the same cytokine present at high levels in serum and in infected tissue is unsure, although it appears to be related to an inflammatory response (91). The ELISpot—IFN-γ test has been used to confirm the induction of cell-mediated immunity in vaccinated dogs. PBMCs are stimulated with total leishmanial soluble antigens—not just the antigens included in the vaccine (56).

### Canine Macrophage Leishmanicidal Assay—CMLA

This is a complex assay performed *in vitro* to demonstrate that the cell-mediated immune response elicited is fully functional and can eliminate the parasite; it reveals the capacity of *Leishmania*-specific T cell clones to induce the leishmanicidal activity of infected macrophages when cultured together. This activity is measured via the reduction in the number of parasites present in cells after 72 h of co-culture. The test can be combined with analyses of the expression of factors such as IFN-γ, NO or iNOS which are directly involved in this leishmanicidal activity (76). This strategy has been used in laboratory studies to confirm immunogenicity after vaccination in dogs, but it is very difficult to use under field conditions (56, 80).

### The Whole Blood Stimulation Assay—WBA

This recently developed IGRA-type test can be used to assess asymptomatic *Leishmania* infection in humans; the results are comparable to those provided by CPA (92). A peripheral blood sample is stimulated with leishmanial antigens and the cytokines/chemokines presents in the plasma determined after 24 h of incubation. IL-2, IFN-γ, IP-10, MIG, and MCP-1 are all associated with a protective immune response (93–95). In humans, this test has been used to confirm full recovery after treatment (94, 96). Our own epidemiological studies involving dogs have confirmed that this is an easy-to-use, robust field technique, and that it can be used to detect natural asymptomatic infection in dogs. More importantly, it can also be used to assess the *Leishmania*-specific immunity induced by vaccination (97). The ease of use of this assay makes it appropriate to veterinary clinical practices for determining the level of protection induced by the vaccination against CVL (98). Further, it can be used to analyze vaccination-induced phenotypic changes in circulating immune cells; the increased expression of Toll-like receptors, activation and co-stimulatory molecules, and of inflammation-associated intracytoplasmic cytokines in neutrophils, monocytes and lymphocytes, have all been reported in Leishmune-immunized dogs (99).

## Concluding Remarks and Perspectives

CVL is a growing public health concern whose control requires the use of effective measures to prevent infection and the development of the disease. Vaccines for CVL represent an important advance for this control, but the complexity of the protective response that these vaccines have to induce in the host makes it difficult their obtaining and the assessment of their efficacy. The techniques discussed for assessing cell-mediated immunity in humans and dogs have all demonstrated their usefulness in this respect and should be used in order to confirm whether a dog has become protected after vaccination. Tools for testing specific immunity against CVL are important given that different vaccines for CVL are on the market and others are in the pipeline. Comparisons between already registered vaccines should go beyond confirming negative serological and parasitological results, but take advantage of cell-mediated immunity tests. The latter should be used in the different phases of clinical development of CVL vaccines and be incorporated into the follow-up of vaccinated animals involved, and into Phase IV post-marketing trials.

## Author Contributions

The author confirms being the sole contributor of this work and has approved it for publication.

### Conflict of Interest Statement

The author declares that the research was conducted in the absence of any commercial or financial relationships that could be construed as a potential conflict of interest.

## References

[1] Alvar J Vélez ID Bern C Herrero M Desjeux P Cano J. Leishmaniasis worldwide and global estimates of its incidence PLoS ONE. (2012) 7:e35671. 10.1371/journal.pone.0035671 22693548 PMC3365071

[2] Gradoni L. Epidemiological surveillance of leishmaniasis in the European Union: operational and research challenges. Euro Surveill. (2013) 18:20539. 10.2807/1560-7917.ES2013.18.30.20539 23929176

[3] Leça Júnior NF Guedes PE Santana LN Almeida Vdos A Carvalho FS Albuquerque GR Epidemiology of canine leishmaniasis in southern Bahia, Brazil. Acta Trop. (2015) 148:115–9. 10.1016/j.actatropica.2015.04.008 25917715

[4] Leite RS Souza NA Barbosa AD Ferreira AL de Andrade AS. Evaluation of conjunctival swab as a mass-screening tool for molecular diagnosis of canine visceral leishmaniasis. Parasitol Res. (2015) 114:2255–62. 10.1007/s00436-015-4418-y 25782681

[5] Moreno J Alvar J. Canine leishmaniasis: epidemiological risk and the experimental model. Trends Parasitol. (2002) 18:399–405. 10.1016/S1471-4922(02)02347-4 12377257

[6] Belo VS Werneck GL Barbosa DS Simões TC Nascimento BW da Silva ES. Factors associated with visceral leishmaniasis in the Americas: a systematic review and meta-analysis. PLoS Negl Trop Dis. (2013) 7:e2182. 10.1371/journal.pntd.0002182 23638203 PMC3636096

[7] WHO Expert Committee on the Control of the Leishmaniases. Control of the Leishmaniases: Report of a Meeting of the WHO Expert Committee on the Control of Leishmaniases, Geneva, 22-26 March 2010. WHO technical report series. Geneva: World Health Organization, (2010) p. 186.

[8] WHO Regional Office for Europe Manual on Case Management and Surveillance of the Leishmaniases in the WHO European Region. (2017). Available online at: https://www.who.int/leishmaniasis/resources/978-92-89052-51-1/en/

[9] Ferroglio E Maroli M Gastaldo S Mignone W Rossi L. Canine leishmaniasis, Italy. Emerg Infect Dis. (2005) 11:1618–20. 10.3201/eid1110.040966 16318709 PMC3366729

[10] Maroli M Rossi L Baldelli R Capelli G Ferroglio E Genchi C. The northward spread of leishmaniasis in Italy: evidence from retrospective and ongoing studies on the canine reservoir and phlebotomine vectors. Trop Med Int Health. (2008) 13:256–64. 10.1111/j.1365-3156.2007.01998.x 18304273

[11] Cruz I Acosta L Gutiérrez MN Nieto J Cañavate C Deschutter J. A canine leishmaniasis pilot survey in an emerging focus of visceral leishmaniasis: posadas (Misiones, Argentina). BMC Infect Dis. (2010) 10:342. 10.1186/1471-2334-10-342 21122107 PMC3002360

[12] PAHO. LEISHMANIASIS. Epidemiological Report of the Americas. (2019). Available online at: http://iris.paho.org/xmlui/bitstream/handle/123456789/50505/Leishreport2019_eng.pdf?ua=1 (accessed March 2019).

[13] Bezerra JMT de Araújo VEM Barbosa DS Martins-Melo FR Werneck GL Carneiro M. Burden of leishmaniasis in Brazil and federated units, 1990-2016: findings from global burden of disease study 2016. PLoS Negl Trop Dis. (2018) 12:e0006697. 10.1371/journal.pntd.0006697 30188898 PMC6126835

[14] Alvar J Cañavate C Molina R Moreno J Nieto J. Canine leishmaniasis. Adv Parasitol. (2004) 57:1–88 10.1016/S0065-308X(04)57001-X 15504537

[15] Reis AB Giunchetti RC Carrillo E Martins-Filho OA Moreno J. Immunity to Leishmania and the rational search for vaccines against canine leishmaniasis. Trends Parasitol. (2010) 26:341–9. 10.1016/j.pt.2010.04.005 20488751

[16] Velez R Ballart C Domenech E Abras A Fernández-Arévalo A Gómez SA. Seroprevalence of canine Leishmania infantum infection in the Mediterranean region and identification of risk factors: the example of North-Eastern and Pyrenean areas of Spain. Prev Vet Med. (2019) 162:67–75. 10.1016/j.prevetmed.2018.10.015 30621900

[17] Solano-Gallego L Morell P Arboix M Alberola J Ferrer L. Prevalence of Leishmania infantum infection in dogs living in an area of canine leishmaniasis endemicity using PCR on several tissues and serology. J Clin Microbiol. (2001) 39:560–3. 10.1128/JCM.39.2.560-563.2001 11158106 PMC87775

[18] Dantas-Torres F Brandão-Filho SP. Visceral leishmaniasis in Brazil: revisiting paradigms of epidemiology and control. Rev Inst Med Trop Sao Paulo. (2006) 48:151–6. 10.1590/S0036-46652006000300007 16847505

[19] Marcondes M Day MJ. Current status and management of canine leishmaniasis in Latin America. Res Vet Sci. (2019) 123:261–72. 10.1016/j.rvsc.2019.01.022 30708238

[20] Silva RAE Andrade AJ Quint BB Raffoul GES Werneck GL Rangel EF. Effectiveness of dog collars impregnated with 4% deltamethrin in controlling visceral leishmaniasis in Lutzomyia longipalpis (Diptera: Psychodidade: Phlebotominae) populations. Mem Inst Oswaldo Cruz. (2018) 113:e170377. 10.1590/0074-02760170377 29590235 PMC5868867

[21] Miró G. Petersen C Cardoso L Bourdeau P Baneth G Solano-Gallego L. Novel areas for prevention and control of canine leishmaniosis. Trends Parasitol. (2017) 33:718–30. 10.1016/j.pt.2017.05.005 28601528

[22] Lopes EG Sevá AP Ferreira F Nunes CM Keid LB Hiramoto RM. Vaccine effectiveness and use of collar impregnated with insecticide for reducing incidence of Leishmania infection in dogs in an endemic region for visceral leishmaniasis, in Brazil. Epidemiol Infect. (2018) 146:401–6. 10.1017/S0950268817003053 29345601 PMC9134555

[23] Reithinger R Coleman PG Alexander B Vieira EP Assis G Davies CR. Are insecticide-impregnated dog collars a feasible alternative to dog culling as a strategy for controlling canine visceral leishmaniasis in Brazil? Int J Parasitol. (2004) 34:55–62. 10.1016/j.ijpara.2003.09.006 14711590

[24] Tarleton RL Pearce EJ Overview of parasitic pathogens. In: Kaufmann SHE Rouse BT Sacks DL editors. The Immune Response to Infection. Washington DC: ASM Press, (2011).

[25] Sher A Immunoparasitology: the making of a modern immunological science. In: Lamb TJ editor. Immunity to Parasitic Infection. West Sussex: John Wiley and Sons, (2012).

[26] Sher A Coffman RL. Regulation of immunity to parasites by T cells and T cell-derived cytokines. Annu Rev Immunol. (1992) 10:385–409. 10.1146/annurev.iy.10.040192.002125 1590992

[27] Müller I Kropf P Kinetoplastids: leishmania. In: Lamb TJ editor. Immunity to Parasitic Infection. West Sussex: John Wiley and Sons, (2012).

[28] Scott P Riley EM Acquired immunity to intracellular protozoa. In Kaufmann S Rouse B Sacks D editors. The Immune Response to Infection. Washington, DC: ASM Press, (2011).

[29] Mansfield JM Olivier M Immune evasion by parasites. In: Kaufmann SHE Rouse BT Sacks DL editors. The Immune Response to Infection, Washington DC: ASM Press, (2011).

[30] Stäger S Joshi T Bankoti R. Immune evasive mechanisms contributing to persistent Leishmania donovani infection. Immunol Res. (2010) 47:14–24. 10.1007/s12026-009-8135-4 20087685

[31] Ontoria E Hernández-Santana YE González-García AC López MC Valladares B Carmelo E. Transcriptional profiling of immune-related genes in leishmania infantum-infected mice: identification of potential biomarkers of infection and progression of disease. Front Cell Infect Microbiol. (2018) 8:197. 10.3389/fcimb.2018.00197 30013952 PMC6036295

[32] Maia C Campino L. Biomarkers Associated With Leishmania infantum Exposure, Infection, and Disease in Dogs. Front Cell Infect Microbiol. (2018) 8:302. 10.3389/fcimb.2018.00302 30237985 PMC6136405

[33] Iborra S Solana JC Requena JM Soto M Vaccine candidates against leishmanial under current research. Expert Rev Vaccines. (2018) 17:323–34. 10.1080/14760584.2018.1459191 29589966

[34] Sacks DL Lal SL Shrivastava SN Blackwell J Neva FA. An analysis of T cell responsiveness in Indian kala-azar. J Immunol. (1987) 138:908–13. 3100620

[35] Carvalho EM Teixeira RS Johnson WD Jr. Cell-mediated immunity in American visceral leishmaniasis: reversible immunosuppression during acute infection. Infect Immun. (1981) 33:498–500. 7275314 10.1128/iai.33.2.498-500.1981 PMC350726

[36] Cabral M O'Grady JE Gomes S Sousa JC Thompson H Alexander J. The immunology of canine leishmaniosis: strong evidence for a developing disease spectrum from asymptomatic dogs. Vet Parasitol. (1998) 76:173–80. 10.1016/S0304-4017(97)00208-2 9615951

[37] Dantas-Torres F Otranto D. Best practices for preventing vector-borne diseases in dogs and humans. Trends Parasitol. (2016) 32:43–55. 10.1016/j.pt.2015.09.004 26507152

[38] Handman E. Leishmaniasis: current status of vaccine development. Clin Microbiol Rev. (2001) 14:229–43. 10.1128/CMR.14.2.229-243.2001 11292637 PMC88972

[39] Mutiso JM Macharia JC Kiio MN Ichagichu JM Rikoi H Gicheru MM. Development of Leishmania vaccines: predicting the future from past and present experience. J Biomed Res. (2013) 27:85–102. 10.7555/JBR.27.20120064 23554800 PMC3602867

[40] Duthie MS Reed SG. Not all antigens are created equally: progress, challenges, and lessons associated with developing a vaccine for Leishmaniasis. Clin Vaccine Immunol. (2017) 24:e00108–7. 10.1128/CVI.00108-17 28515135 PMC5498718

[41] Mayrink W Genaro O Silva JC da Costa RT Tafuri WL Toledo VP. Phase I and II open clinical trials of a vaccine against Leishmania chagasi infections in dogs. Mem Inst Oswaldo Cruz. (1996) 91:695–7. 10.1590/S0074-02761996000600006 9283646

[42] Mohebali M Khamesipour A Mobedi I Zarei Z Hashemi-Fesharki R Double-blind randomized efficacy field trial of alum precipitated autoclaved Leishmania major vaccine mixed with BCG against canine visceral leishmaniosis in Meshkin-Shahr district, I.R. *Iran*. Vaccine. (2004) 22:4097–100. 10.1016/j.vaccine.2004.03.058 15364462

[43] Borja-Cabrera GP Correia Pontes NN da Silva VO Paraguai de Souza E Santos WR Gomes EM. Long lasting protection against canine kala-azar using the FML-QuilA saponin vaccine in an endemic area of Brazil (São Gonçalo do Amarante, RN). Vaccine. (2002) 20:3277–84. 10.1016/S0264-410X(02)00294-3 12213397

[44] Molano I Alonso MG Mirón C Redondo E Requena JM Soto M. A Leishmania infantum multi-component antigenic protein mixed with live BCG confers protection to dogs experimentally infected with, L. infantum Vet Immunol Immunopathol. (2003) 92:1–13. 10.1016/S0165-2427(02)00315-X 12628759

[45] Moreno J Nieto J Masina S Cañavate C Cruz I Chicharro C. Immunization with H1, HASPB1 and MML Leishmania proteins in a vaccine trial against experimental canine leishmaniasis. Vaccine. (2007) 25:5290–300. 10.1016/j.vaccine.2007.05.010 17576026 PMC2695600

[46] Martin V Vouldoukis I Moreno J McGahie D Gueguen S Cuisinier AM. The protective immune response produced in dogs after primary vaccination with the LiESP/QA-21 vaccine (CaniLeish®) remains effective against an experimental challenge one year later. Vet Res. (2014) 45:69. 10.1186/1297-9716-45-69 24964736 PMC4086268

[47] Rafati S Nakhaee A Taheri T Taslimi Y Darabi H Eravani D. Protective vaccination against experimental canine visceral leishmaniasis using a combination of DNA and protein immunization with cysteine proteinases type I and II of, L. infantum. Vaccine. (2005) 23:3716–25. 10.1016/j.vaccine.2005.02.009 15882533

[48] Ramiro MJ Zárate JJ Hanke T Rodriguez D Rodriguez JR Esteban M. Protection in dogs against visceral leishmaniosis caused by Leishmania infantum is achieved by immunization with a heterologous prime-boost regime using DNA and vaccinia recombinant vectors expressing LACK. Vaccine. (2003) 21:2474–84. 10.1016/S0264-410X(03)00032-X 12744881

[49] Daneshvar H Namazi MJ Kamiabi H Burchmore R Cleaveland S Phillips S. Gentamicin-attenuated Leishmania infantum vaccine: protection of dogs against canine visceral leishmaniosis in endemic area of southeast of Iran. PLoS Negl Trop Dis. (2014) 8:e2757. 10.1371/journal.pntd.0002757 24743691 PMC3990512

[50] Gradoni L. Canine Leishmania vaccines: still a long way to go. Vet Parasitol. (2015) 208:94–100. 10.1016/j.vetpar.2015.01.003 25620293

[51] Fiuza JA Gannavaram S Santiago Hda C Selvapandiyan A Souza DM Passos LS. Vaccination using live attenuated Leishmania donovani centrin deleted parasites induces protection in dogs against Leishmania infantum. Vaccine. (2015) 33:280–8. 10.1016/j.vaccine.2014.11.039 25475955

[52] Shahbazi M Zahedifard F Taheri T Taslimi Y Jamshidi S Shirian S. Evaluation of live recombinant nonpathogenic leishmania tarentolae expressing cysteine proteinase and A2 genes as a candidate vaccine against experimental canine visceral leishmaniasis. PLoS ONE. (2015) 10:e0132794. 10.1371/journal.pone.0132794 26197085 PMC4509652

[53] Barati M Mohebali M Alimohammadian MH Khmesipour A Keshavarz H Akhoundi B. Double-blind randomized efficacy field trial of alum precipitated autoclaved leishmania major (Alum-ALM) vaccine mixed with bcg plus imiquimod vs. placebo control group. Iran J Parasitol. (2015) 10,351–359. 26622290 PMC4662735

[54] Regina-Silva S Feres AM França-Silva JC Dias ES Michalsky ÉM de Andrade HM. Field randomized trial to evaluate the efficacy of the Leish-Tec® vaccine against canine visceral leishmaniasis in an endemic area of Brazil. Vaccine. (2016) 34:2233–9. 10.1016/j.vaccine.2016.03.019 26997002

[55] Grimaldi G Jr Teva A Dos-Santos CB Santos FN Pinto ID Fux B. Field trial of efficacy of the Leish-tec® vaccine against canine leishmaniasis caused by Leishmania infantum in an endemic area with high transmission rates. PLoS ONE. (2017) 12:e0185438. 10.1371/journal.pone.0185438 28953944 PMC5617193

[56] Moreno J Vouldoukis I Martin V McGahie D Cuisinier AM Gueguen S. Use of a LiESP/QA-21 vaccine (CaniLeish) stimulates an appropriate Th1-dominated cell-mediated immune response in dogs. PLoS Negl Trop Dis. (2012) 6:e1683. 10.1371/journal.pntd.0001683 22724031 PMC3378610

[57] Carcelén J Iniesta V Fernández-Cotrina J Serrano F Parejo JC Corraliza I. The chimerical multi-component Q protein from Leishmania in the absence of adjuvant protects dogs against an experimental Leishmania infantum infection. Vaccine. (2009) 27:5964–73. 10.1016/j.vaccine.2009.07.069 19666153

[58] Oliva G Nieto J Foglia Manzillo V Cappiello S Fiorentino E Di Muccio T. A randomised, double-blind, controlled efficacy trial of the LiESP/QA-21 vaccine in naïve dogs exposed to two Leishmania infantum transmission seasons. PLoS Negl Trop Dis. (2014) 8:e3213. 10.1371/journal.pntd.0003213 25299614 PMC4191955

[59] Fernández Cotrina J Iniesta V Monroy I Baz V Hugnet C Marañon F. A large-scale field randomized trial demonstrates safety and efficacy of the vaccine LetiFend® against canine leishmaniosis. Vaccine. (2018) 36:1972–82. 10.1016/j.vaccine.2018.02.111 29525281

[60] Testasicca MC dos Santos MS Machado LM Serufo AV Doro D Avelar D. Antibody responses induced by Leish-Tec®, an A2-based vaccine for visceral leishmaniasis, in a heterogeneous canine population. Vet Parasitol. (2014) 204:169–76. 10.1016/j.vetpar.2014.04.025 24863572

[61] Murray HW Oca MJ Granger AM Schreiber RD. Requirement for T cells and effect of lymphokines in successful chemotherapy for an intracellular infection. Experimental visceral leishmaniasis. J Clin Invest. (1989) 83:1253–7. 10.1172/JCI114009 2539396 PMC303815

[62] Solano-Gallego L Llull J Ramis A Fernández-Bellon H Rodríguez A Ferrer L. Longitudinal study of dogs living in an area of Spain highly endemic for leishmaniasis by serologic analysis and the leishmanin skin test. Am J Trop Med Hyg. (2005) 72:815–8. 10.4269/ajtmh.2005.72.815 15964969

[63] Ordeix L Silva JEDS Llull J Quirola P Montserrat-Sangrà S. Martínez-Orellana P. Histological and Immunological Description of the Leishmanin Skin Test in Ibizan Hounds. J Comp Pathol. (2018) 158:56–65. 10.1016/j.jcpa.2017.11.004 29422316

[64] Leandro C Santos-Gomes GM Campino L Romão P Cortes S Rolão N. Cell mediated immunity and specific IgG1 and IgG2 antibody response in natural and experimental canine leishmaniosis. Vet Immunol Immunopathol. (2001) 79:273–84. 10.1016/S0165-2427(01)00270-7 11389961

[65] Quinnell RJ Courtenay O Davidson S Garcez L Lambson B Ramos P. Detection of Leishmania infantum by PCR, serology and cellular immune response in a cohort study of Brazilian dogs. Parasitology. (2001) 122:253–61. 10.1017/S0031182001007363 11289062

[66] Cardoso L Neto F Sousa JC Rodrigues M Cabral M. Use of a leishmanin skin test in the detection of canine Leishmania-specific cellular immunity. Vet Parasitol. (1998) 79:213–20. 10.1016/S0304-4017(98)00169-1 9823061

[67] Solano-Gallego L Llull J Ramos G Riera C Arboix M Alberola J. The Ibizian hound presents a predominantly cellular immune response against natural Leishmania infection. Vet Parasitol. (2000) 90:37–45. 10.1016/S0304-4017(00)00223-5 10828510

[68] Fernández-Bellon H Solano-Gallego L Rodríguez A Rutten VP Hoek A Ramis A. Comparison of three assays for the evaluation of specific cellular immunity to Leishmania infantum in dogs. Vet Immunol Immunopathol. (2005) 107:163–9. 10.1016/j.vetimm.2005.04.002 15922459

[69] Rodríguez-Cortés A Fernández-Bellón H Ramis A Ferrer L Alberola J Solano-Gallego L. Leishmania-specific isotype levels and their relationship with specific cell-mediated immunity parameters in canine leishmaniasis. Vet Immunol Immunopathol. (2007) 116:190–8. 10.1016/j.vetimm.2007.01.015 17321600

[70] Dos-Santos WL Jesus EE Paranhos-Silva M Pereira AM Santos JC Baleeiro CO. Associations among immunological, parasitological and clinical parameters in canine visceral leishmaniasis: emaciation, spleen parasitism, specific antibodies and leishmanin skin test reaction. Vet Immunol Immunopathol. (2008) 123:251–9. 10.1016/j.vetimm.2008.02.004 18359091

[71] Chamakh-Ayari R Bras-Gonçalves R Bahi-Jaber N Petitdidier E Markikou-Ouni W Aoun K. *In vitro* evaluation of a soluble Leishmania promastigote surface antigen as a potential vaccine candidate against human leishmaniasis. PLoS ONE. (2014) 9:e92708. 10.1371/journal.pone.0092708 24786587 PMC4008367

[72] Rodríguez-Cortés A Ojeda A López-Fuertes L Timón M Altet L Solano-Gallego L. A long term experimental study of canine visceral leishmaniasis. Int J Parasitol. (2007) 37:683–93 10.1016/j.ijpara.2006.11.007 17239885

[73] Moreno J Nieto J Chamizo C González F Blanco F Barker DC. The immune response and PBMC subsets in canine visceral leishmaniasis before, and after, chemotherapy. Vet Immunol Immunopathol. (1999) 71:181–95. 10.1016/S0165-2427(99)00096-3 10587300

[74] Carrillo E Ahmed S Goldsmith-Pestana K Nieto J Osorio Y Travi B. Immunogenicity of the P-8 amastigote antigen in the experimental model of canine visceral leishmaniasis. Vaccine. (2007) 25:1534–43. 10.1016/j.vaccine.2006.10.036 17178178 PMC2571115

[75] Carrillo E Crusat M Nieto J Chicharro C Thomas Mdel C Martínez E. Immunogenicity of HSP-70, KMP-11 and PFR-2 leishmanial antigens in the experimental model of canine visceral leishmaniasis. Vaccine. (2008) 26:1902–11. 10.1016/j.vaccine.2008.01.042 18321614

[76] Petitdidier E Pagniez J Papierok G Vincendeau P Lemesre JL Bras-Gonçalves R. Recombinant forms of leishmania amazonensis excreted/secreted promastigote surface antigen (PSA) induce protective immune responses in dogs. PLoS Negl Trop Dis. (2016) 10:e0004614. 10.1371/journal.pntd.0004614 27223609 PMC4880307

[77] Roatt BM Aguiar-Soares RD Vitoriano-Souza J Coura-Vital W Braga SL Corrêa-Oliveira R. Performance of LBSap vaccine after intradermal challenge with, L. infantum and saliva of Lu longipalpis: immunogenicity and parasitological evaluation. PLoS ONE. (2012) 7:e49780. 10.1371/journal.pone.0049780 23189161 PMC3506642

[78] Aguiar-Soares RD Roatt BM Ker HG Moreira ND Mathias FA Cardoso JM. LBSapSal-vaccinated dogs exhibit increased circulating T-lymphocyte subsets (CD4^+^ and CD8^+^) as well as a reduction of parasitism after challenge with Leishmania infantum plus salivary gland of Lutzomyia longipalpis. Parasit Vectors. (2014) 7:61. 10.1186/1756-3305-7-61 24507702 PMC3943450

[79] Mendonça L de Resende LA Lanna MF Aguiar-Soares RD Roatt BM. Multicomponent LBSap vaccine displays immunological and parasitological profiles similar to those of Leish-Tec® and Leishmune® vaccines against visceral leishmaniasis. Parasit Vectors. (2016) 9:472. 10.1186/s13071-016-1752-6 27577735 PMC5006379

[80] Moreno J Vouldoukis I Schreiber P Martin V McGahie D Gueguen S. Primary vaccination with the LiESP/QA-21 vaccine (CaniLeish) produces a cell-mediated immune response which is still present 1 year later. Vet Immunol Immunopathol. (2014) 158:199–207. 10.1016/j.vetimm.2014.01.011 24560650

[81] Araújo MS de Andrade RA Sathler-Avelar R Magalhães CP Carvalho AT Andrade MC. Immunological changes in canine peripheral blood leukocytes triggered by immunization with first or second generation vaccines against canine visceral leishmaniasis. Vet Immunol Immunopathol. (2011) 141:64–75. 10.1016/j.vetimm.2011.02.007 21439654

[82] Resende LA Roatt BM Aguiar-Soares RD Viana KF Mendonça LZ Lanna MF. Cytokine and nitric oxide patterns in dogs immunized with LBSap vaccine, before and after experimental challenge with Leishmania chagasi plus saliva of Lutzomyia longipalpis. Vet Parasitol. (2013) 198:371–81. 10.1016/j.vetpar.2013.09.011 24129068 PMC7115768

[83] Resende LA Aguiar-Soares RD Gama-Ker H Roatt BM Mendonça LZ Alves ML. Impact of LbSapSal vaccine in canine immunological and parasitological features before and after Leishmania chagasi-Challenge. PLoS ONE. (2016) 11:e0161169. 10.1371/journal.pone.0161169 27556586 PMC4996460

[84] Abeijon C Daifalla N Krautz-Peterson G Pizzirani S Beamer G Frazatti-Gallina NM. Immunogenicity in dogs and protection against visceral leishmaniasis induced by a 14kDa Leishmania infantum recombinant polypeptide. Trials Vaccinol. (2016) 5:1–7. 10.1016/j.trivac.2015.11.001 26640609 PMC4667363

[85] Nylén S Khamesipour A Mohammadi A Jafari-Shakib R Eidsmo L Noazin S. Surrogate markers of immunity to Leishmania major in leishmanin skin test negative individuals from an endemic area re-visited. Vaccine. (2006) 24:6944–695 10.1016/j.vaccine.2006.05.016 17049693

[86] Liew FY. Regulation of nitric oxide synthesis in infectious and autoimmune diseases. Immunol Lett. (1994) 43:95–8. 10.1016/0165-2478(94)00157-X 7737695

[87] Moll H Berberich C. Dendritic cell-based vaccination strategies: induction of protective immunity against leishmaniasis. Immunobiology. (2001) 204:659–66. 10.1078/0171-2985-00105 11846231

[88] Chamizo C Moreno J Alvar J. Semi-quantitative analysis of cytokine expression in asymptomatic canine leishmaniasis. Vet Immunol Immunopathol. (2005) 103:67–75. 10.1016/j.vetimm.2004.08.010 15626462

[89] Manna L Reale S Viola E Vitale F Foglia Manzillo V Pavone LM. Leishmania DNA load and cytokine expression levels in asymptomatic naturally infected dogs. Vet Parasitol. (2006) 142:271–80 10.1016/j.vetpar.2006.06.028 16920264

[90] Araújo MS de Andrade RA Sathler-Avelar R Teixeira-Carvalho A Andrade MC Vianna LR. T-cell-derived cytokines, nitric oxide production by peripheral blood monocytes and seric anti-Leishmania (Leishmania) chagasi IgG subclass patterns following immunization against canine visceral leishmaniasis using Leishvaccine and Leishmune. Vaccine. (2009) 27:1008–17. 10.1016/j.vaccine.2008.11.104 19110023

[91] Cillari E Vitale G Arcoleo F D'Agostino P Mocciaro C Gambino G. *In vivo* and *in vitro* cytokine profiles and mononuclear cell subsets in Sicilian patients with active visceral leishmaniasis. Cytokine. (1995) 7:740–745. 10.1006/cyto.1995.0088 8580385

[92] Carrillo E Carrasco-Antón N López-Medrano F Salto E Fernández L San Martín JV. Cytokine release assays as tests for exposure to leishmania, and for confirming cure from leishmaniasis, in solid organ transplant recipients. PLoS Negl Trop Dis. (2015) 9:e0004179. 10.1371/journal.pntd.0004179 26496365 PMC4619795

[93] Ibarra-Meneses AV Carrillo E Sánchez C García-Martínez J López Lacomba D San Martin JV. Interleukin-2 as a marker for detecting asymptomatic individuals in areas where Leishmania infantum is endemic. Clin Microbiol Infect. (2016) 22:739.e1–4. 10.1016/j.cmi.2016.05.021 27265372

[94] Ibarra-Meneses AV Ghosh P Hossain F Chowdhury R Mondal D Alvar J. IFN-γ, IL-2, IP-10, and MIG as Biomarkers of Exposure to Leishmania spp., and of Cure in Human Visceral Leishmaniasis. Front Cell Infect Microbiol. (2017) 7:200. 10.3389/fcimb.2017.00200 28620584 PMC5449718

[95] Ibarra-Meneses AV Mondal D Alvar J Moreno J Carrillo E. Cytokines and chemokines measured in dried SLA-stimulated whole blood spots for asymptomatic Leishmania infantum and Leishmania donovani infection. Sci Rep. (2017) 7:17266. 10.1038/s41598-017-17315-z 29222521 PMC5722824

[96] Ibarra-Meneses AV Sanchez C Alvar J Moreno J Carrillo E. Monocyte chemotactic protein 1 in plasma from soluble leishmania antigen-stimulated whole blood as a potential biomarker of the cellular immune response to leishmania infantum. Front Immunol. (2017) 8:1208. 10.3389/fimmu.2017.01208 29033933 PMC5626820

[97] Paixao Seva A Ferreira F Lopez Gallucci E Soares Martins R Carrillo E Moreno J Immunityresponse in vaccinated dogs against leishmaniasis in a Brazilian endemic area. In: 6th World Congress on Leishmaniasis, Toledo, (2017).

[98] Costa-Pereira C Moreira ML Soares RP Marteleto BH Ribeiro VM França-Dias MH. One-year timeline kinetics of cytokine-mediated cellular immunity in dogs vaccinated against visceral leishmaniasis. BMC Vet Res. (2015) 11:92. 10.1186/s12917-015-0397-6 25880646 PMC4405846

[99] Moreira ML Costa-Pereira C Alves ML Marteleto BH Ribeiro VM Peruhype-Magalhães V. Vaccination against canine leishmaniosis increases the phagocytic activity, nitric oxide production and expression of cell activation/migration molecules in neutrophils and monocytes. Vet Parasitol. (2016) 220:33–45. 10.1016/j.vetpar.2016.02.009 26995719

